# Case Report: Rare invasive aspergillosis with brain abscess in a non–classically immunosuppressed patient, and pooled analysis of individual patient data (2000–2024)

**DOI:** 10.3389/fsurg.2025.1674057

**Published:** 2025-10-23

**Authors:** Moksada Regmi, Shikun Liu, Yuwei Dai, Jingyi Ye, Xiaodong Chen, Jun Yang, Chenlong Yang

**Affiliations:** 1 State Key Laboratory of Vascular Homeostasis and Remodeling, Department of Neurosurgery, Peking University Third Hospital, Peking University, Beijing, China; 2 Center for Precision Neurosurgery and Oncology of Peking University Health Science Center, Peking University, Beijing, China; 3 Peking University Health Science Center, Beijing, China; 4 Center for Oculocranial Pressure Instability Disorders (COPID), Henan Academy of Innovations in Medical Science (AIMS), Zhengzhou, China; 5 Peking University School of Economics, Beijing, China

**Keywords:** aspergillosis, brain abscess, non-classically immunosuppressed, voriconazole, isavuconazole

## Abstract

Intracranial aspergillosis is uncommon but often lethal, especially in classically immunocompromised hosts. We report a 71-year-old man with poorly controlled diabetes (a non-classical risk factor) who developed bilateral frontal abscesses due to *Aspergillus fumigatus*. After an initial craniotomy with negative cultures and galactomannan, recurrent disease was confirmed by stereotactic biopsy with next-generation sequencing (NGS). Targeted azole therapy (voriconazole, isavuconazole) and multidisciplinary care led to marked clinical and radiographic improvement. We also pooled 343 published cases (2000–2024): overall mortality was 34.6%, and 21.8% among patients without classical immunosuppression (including some with non-classical factors such as diabetes). Improved survival in recent decades likely reflects earlier diagnosis and broader azole use, though inference is limited by case-based evidence. Early tissue diagnosis (including molecular testing), timely surgery when indicated, and CNS-penetrant azoles can yield favorable outcomes in non-classically immunosuppressed patients.

## Background

Intracranial *Aspergillus* infection is a severe complication of invasive aspergillosis, primarily affecting immunocompromised patients (e.g., those with hematologic malignancies, transplant recipients, or on chronic corticosteroids) (1–5). Historically, reported mortality rates for intracranial aspergillosis were as high as 85% to 99%, especially in immunocompromised hosts, and when complicated by brain abscesses, the rate reportedly approaches 100% in such patients (6–14).

*Aspergillus* typically reaches the brain through hematogenous spread from a primary pulmonary focus or by direct extension from the paranasal sinuses (10, 15). The clinical manifestations of cerebral aspergillosis are often nonspecific, leading to delayed diagnosis (16, 17). Common symptoms include headache, altered mental status, focal neurological deficits, seizures, and visual disturbances. In patients without classical immunosuppression but with non-classical risk factors (e.g., diabetes), the course may be more indolent and may present as meningitis or granulomatous mass (18).

Intracranial aspergillosis is an exceedingly rare condition, even more so among immunocompetent patients. The existing literature primarily comprises single case reports or small retrospective studies, which provide insufficient data for robust prognostic assessments. Over the last 20–25 years, medical diagnostics and treatments have improved significantly. To contextualize our case, we compiled a pooled individual-patient analysis of reports since 2000, explicitly comparing outcomes by immune status. For terminology consistency throughout, we use “non-classically immunosuppressed” to denote patients lacking classical immunosuppressive conditions (e.g., no hematologic malignancy, transplant, HIV/AIDS, active chemotherapy, prolonged high-dose steroids, or primary immunodeficiency) but who carry risk modifiers such as diabetes.

## Case presentation

A 71-year-old man with a 17-year history of poorly controlled type 2 diabetes (HbA1c 9.5%) underwent endoscopic resection of a sphenoid sinus mass with sinusotomy for chronic sinusitis (15 months before definitive diagnosis). Histopathology showed inflammation without fungi, and symptoms initially improved. Six months later, he developed aphasia, cognitive slowing, and personality change without fever. MRI at a local hospital suggested bilateral frontal abscesses; mannitol partially improved speech.

**Figure 1:**
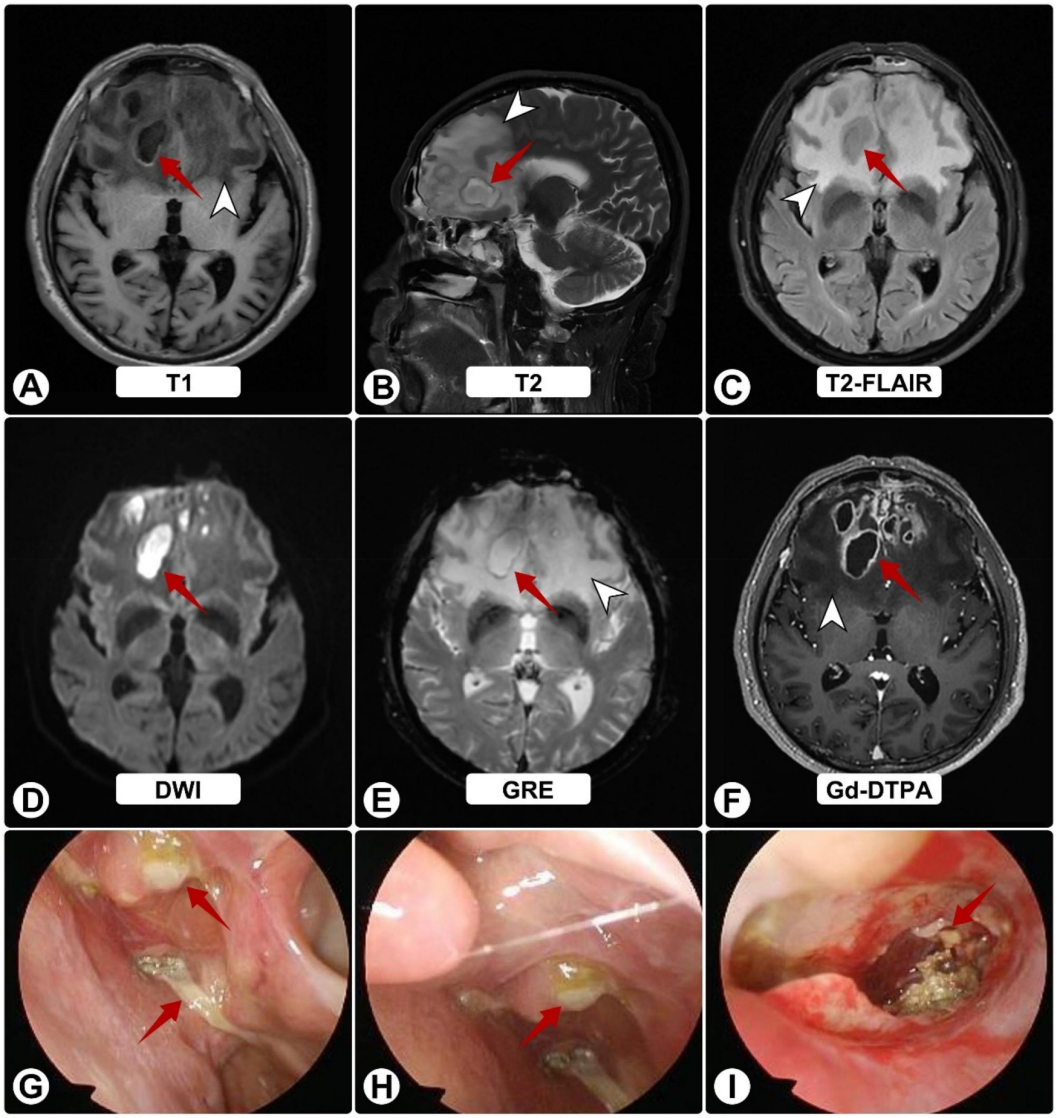
Brain MRI and nasal endoscopy on admission. **(A–F)** White arrowheads indicate surrounding edema; red arrows show abscesses. MRI shows bilateral frontal lesions appearing hyperintense on T1 **(A)** and T2 **(B)**, and isointense on FLAIR **(C)** Post-contrast images **(D–F)** reveal ring enhancement of lesions with extensive vasogenic edema and midline shift. **(G–I)** Nasal endoscopy shows purulent sinus secretions (red arrows).

Two months thereafter he presented to our center. MRI demonstrated bilateral frontal lesions with extensive edema and ring enhancement (Figures 1A–F); nasal endoscopy confirmed purulence (Figures 1G–I). Pre-operative CT showed anterior skull-base sclerosis and erosion consistent with osteomyelitis (Figure 3A). Laboratory tests revealed leukocytosis with neutrophilia and mildly elevated CRP; HIV screening was negative. Serum galactomannan (GM) was negative.

Given encapsulated abscesses with sinus and skull-base involvement, he underwent bilateral frontal craniotomy for evacuation and anterior skull-base reconstruction. Abundant pus was encountered; bacterial, fungal, and mycobacterial cultures and histopathology were negative.

**Figure 2:**
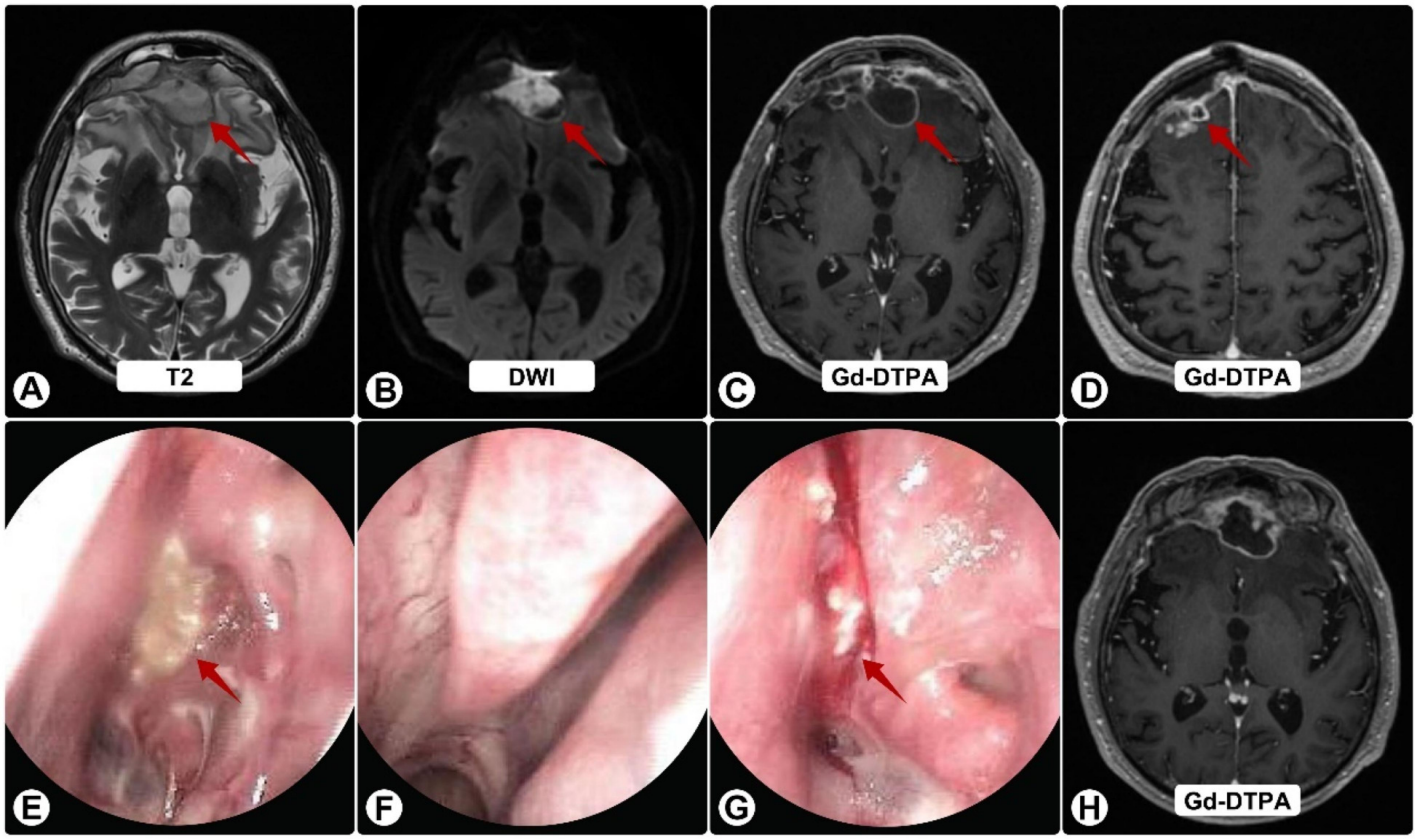
Follow-up brain MRI and nasal endoscopy. **(A)** T2-weighted image showing enlargement of frontal lesions (red arrows) with increased edema. **(B)** DWI shows persistent restricted diffusion. **(C,D,H)** Gd-DTPA–enhanced images demonstrate continued ring enhancement, with some solid components **(H)**, **(E–G)** Endoscopy reveals persistent purulent secretions (red arrows) on the nasal mucosa **(E)**, left middle meatus **(F)**, and sphenoid sinus ostium **(G)**, despite antibiotics.

Postoperatively, he received vancomycin and ceftriaxone plus fluconazole (guided by early nanopore reads suggesting possible *Candida*). He was discharged to complete IV antibiotics and fluconazole. Two weeks later he worsened neurologically. Follow-up MRI showed progression with persistent ring enhancement and diffusion restriction (Figures 2A–D); endoscopy again revealed purulence (Figures 2E–G).

**Figure 3:**
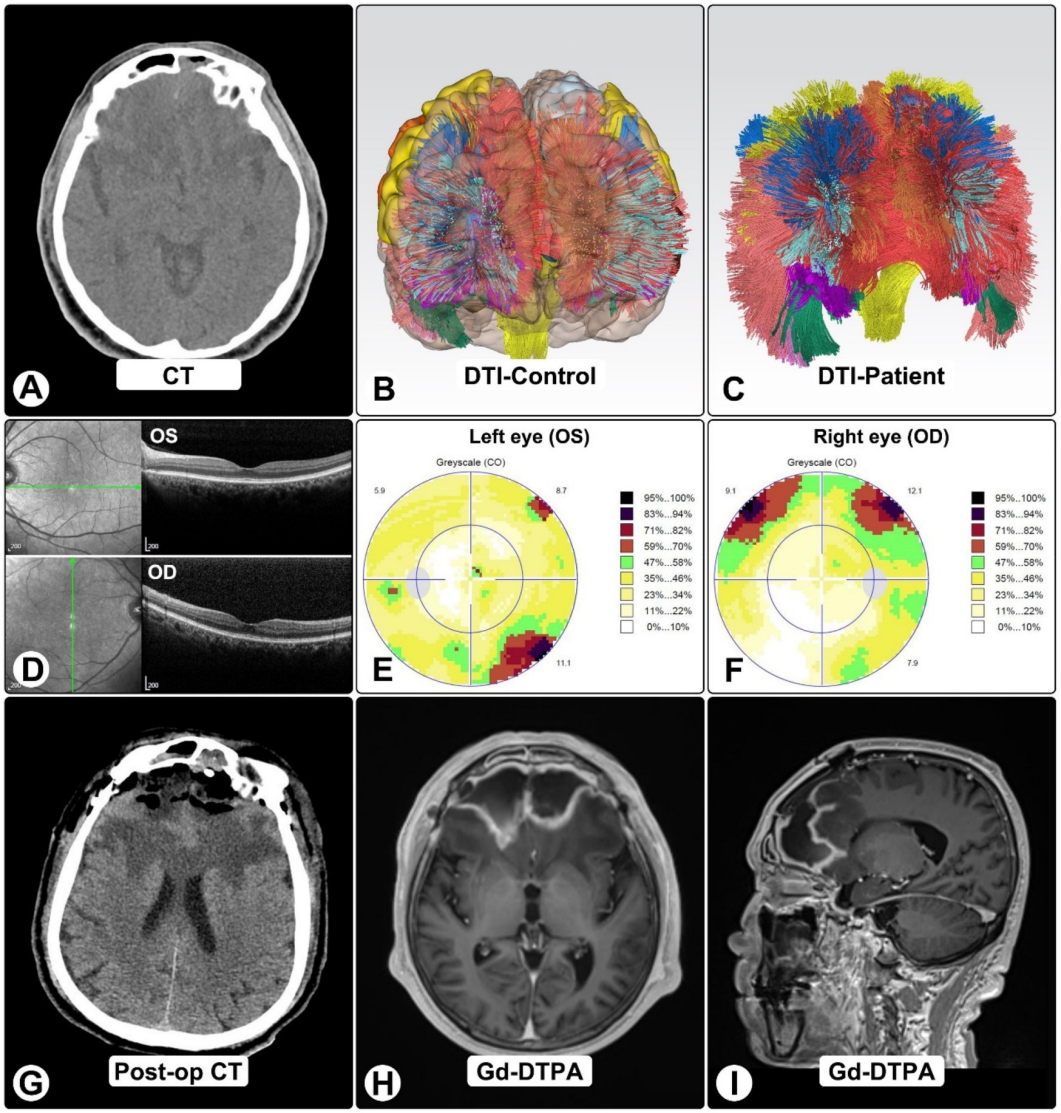
DTI tractography, OCT, and visual findings. **(A)** Pre-op CT shows bilateral frontal lesions with vasogenic edema and mass effect. **(B,C)** DTI tractography in normal brain **(B)** and patient **(C)**, showing marked frontal tract loss, including bilateral anterior thalamic radiations, genu of corpus callosum, cingulum bundles, and forceps minor. **(D)** OCT imaging. **(E,F)** Octopus visual fields show right superior altitudinal defect **(E)** and left inferotemporal scotoma **(F)**, consistent with compressive optic neuropathy. **(G)** Post-op CT showing expected changes after craniotomy and abscess evacuation. **(H,I)** One-month post-op MRI shows residual ring-enhancing lesions with reduced size and mass effect.

Four months after craniotomy, stereotactic biopsy confirmed *A. fumigatus* by NGS; serum GM and β-D-glucan were now positive. Sinonasal biopsies demonstrated invasive hyphae. DTI tractography showed marked frontal tract loss (Figures 3B,C). OCT was normal, while visual fields showed dense bilateral defects consistent with compressive optic neuropathy (Figures 3D–F).

**Figure 4:**
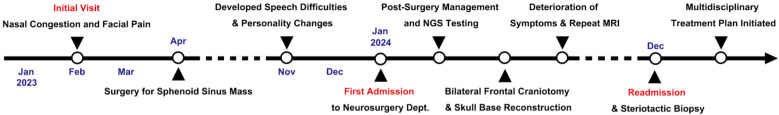
Timeline of clinical events and interventions.

Multidisciplinary management with CNS-penetrant azoles (voriconazole and isavuconazole), blood-pressure control, and supportive care led to steady improvement. One-month MRI showed smaller lesions and reduced mass effect (Figures 3H,I). At six months, he was independent in daily activities with mild residual cognitive impairment and persistent visual-field loss (Figure 4).

## Methods for pooled analysis

### Search strategy and eligibility

**Figure 5:**
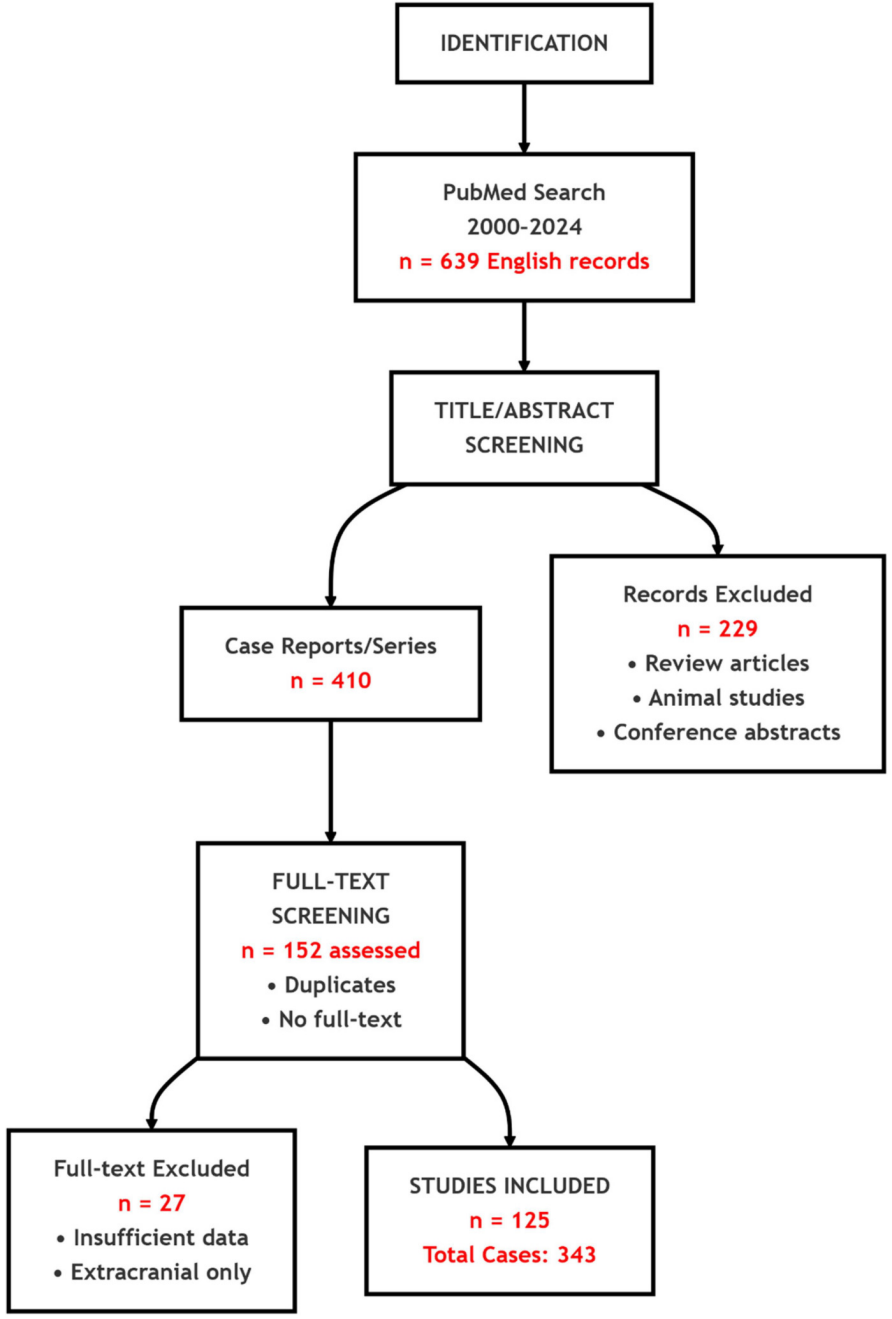
PRISMA flowchart of study selection.

We searched PubMed for English-language reports from January 1, 2000 to May 10, 2024 using (“intracranial aspergillosis” OR “Aspergillus brain abscess” OR “central nervous system Aspergillus” OR “cerebral Aspergillus” OR “brain aspergillosis”). Inclusion required: (1) histopathological, culture, or molecular confirmation of intracranial aspergillosis; (2) radiologic evidence of an intracranial lesion (intraparenchymal or extradural); and (3) documented immune status and outcome. We excluded reviews, animal studies, conference abstracts, studies with insufficient intracranial data, and reports limited to extracranial infection. PRISMA flow: 639 records → 229 excluded at screening; after removing 258 duplicates/inaccessible texts, 152 full texts reviewed; 27 excluded; 125 studies included (343 cases) (Figure 5).

### Terminology

“Classically immunocompromised” comprised hematologic malignancy, solid-organ or stem-cell transplantation, HIV/AIDS, active cytotoxic chemotherapy, prolonged high-dose corticosteroids (>20 mg/day prednisone-equivalent for >2 weeks), or primary immunodeficiency. “Non-classically immunosuppressed” included conditions such as diabetes that alter host defenses without meeting classical criteria. For clarity, we report outcomes for patients “without classical immunosuppression,” which may include non-classical factors.

### Results

Among 343 patients (201 male, 107 female, 35 unspecified), there were 119 deaths, with 11 additional deaths unrelated to aspergillosis; 213 were recovered or stable at last follow-up. Overall mortality was 34.6%; mortality among patients without classical immunosuppression (including some with non-classical factors) was 21.8%. Detailed per-case data are provided in Supplementary Table S1.

## Discussion and conclusion

This case underscores diagnostic pitfalls in intracranial aspergillosis, particularly when early biomarkers are negative. The GM assay has variable sensitivity: at an optical density index of 0.5, pooled sensitivity is ∼82% (≈18% false-negative rate); at 1.0, sensitivity declines to ∼72% (≈28% false-negative rate) (19–21). Thus, negative GM should not preclude biopsy or molecular testing when imaging and clinical evolution are concerning. The eventual detection of *Aspergillus* DNA via NGS on a biopsy specimen was pivotal, enabling targeted antifungal therapy and likely preventing a fatal outcome.

Surgery remains important for encapsulated collections, mass effect, or diagnostic uncertainty, both to decompress and to obtain tissue for definitive identification (culture, histology, PCR/NGS). In our patient, delayed confirmation by NGS ultimately enabled targeted therapy.

Therapeutically, outcomes have improved with CNS-penetrant triazoles—especially voriconazole—compared with amphotericin B or itraconazole, which historically showed poor CNS efficacy and tolerability (22–33). Isavuconazole may offer additional options, as illustrated by this case, though further CNS-specific data are needed (34, 35). Non-classical risk factors (e.g., diabetes), disruption of anatomic barriers (e.g., sinus surgery), and transient immune dysfunction can permit angioinvasion even without classical immunosuppression. Recognizing these scenarios can prompt earlier imaging, biopsy, and azole initiation (36).

In the future, standardization of molecular diagnostics, therapeutic drug monitoring for azoles, and optimized combination/sequencing strategies may further lower mortality; exploratory immunomodulatory approaches merit study but lie beyond the scope of this report.

## Conclusion

Intracranial aspergillosis remains a high-stakes neurosurgical and infectious-disease emergency, yet patients without classical immunosuppression—including those with non-classical risks such as diabetes—can achieve good outcomes when clinicians maintain suspicion despite early false-negative biomarkers, pursue early tissue diagnosis with biopsy/NGS, and combine indicated surgical management with CNS-penetrant azoles (e.g., voriconazole, isavuconazole) under multidisciplinary care.

## Data Availability

The original contributions presented in the study are included in the article/Supplementary Material, further inquiries can be directed to the corresponding authors.
