# Supplementary material for: Case Report: Rare invasive aspergillosis with brain abscess in a non–classically immunosuppressed patient, and pooled analysis of individual patient data (2000–2024)
Source: Front Surg. 2025 Oct 23;12:1674057. doi: 10.3389/fsurg.2025.1674057 (PMC12589800; doi:10.3389/fsurg.2025.1674057)
Supplement: Supplementary file 1 [file Table1.docx]

**Supplementary**

**Supplementary Table S1.** Summary of all studies reporting intracranial aspergillosis in patients without known immunosuppression or underlying comorbidities since 2000 AD.

| Author | Year of Publication | Country | Patient Number | Age | Gender | Underlying Conditions | Clinical Presentation | Diagnostic Method | Treatment | Outcome |
| --- | --- | --- | --- | --- | --- | --- | --- | --- | --- | --- |
| Sagar *et al.* | 2022 | India | 1 | 26 | Male | None | Headache, vomiting | CT, MRI | Surgery, voriconazole | Full recovery |
| Kumar *et al.* | 2021 | India | 1 | 27 | Male | None | Fever, productive cough, hemiparesis | CT, autopsy | Broad-spectrum antibiotics, anti-tuberculosis treatment | Died from illness |
| Elgamal *et al.* | 2020 | Saudi Arabia | 1 | 24 | Male | None | Headache, blurring of vision, vomiting, recurrent seizures, and bilateral gross papilloedema. | CT, MRI | Frontal craniotomy, intravenous and intracavitary amphotericin B, itraconazole | Neurologically intact, seizures controlled |
| Khandelwal *et al.* | 2020 | India | 1 | 2 months | 1 Male | None | Fever, mild left hemiparesis | MRI, surgical excision | Amphotericin B, surgery | Improved, follow-up CT showed no abnormalities |
| Ma *et al.* | 2020 | China | 6 | 20-59 | 4 Male, 2 Female | Immunocompetent | Headache, visual impairment, hemiplegia, epilepsy | MRI, biopsy, CSF culture | Voriconazole | 5 improved, 1 died |
| Joung *et al.* | 2019 | South Korea | 1 | 62 | Female | None | Headache, hemiparesis | MRI, biopsy | Trimethoprim-sulfamethoxazole, itraconazole | Resolution of brain abscess |
| Pant *et al.* | 2019 | India | 1 | 34 | Male | None | Numbness, facial weakness, eye movement restriction | MRI, Histopathology | Surgical resection, not specified if antifungal used | Died on day 6 post-surgery |
| Bora *et al.* | 2019 | India | 18 | 5-65 | 6 Female, 12 Male | Immunocompetent | Headache, vomiting, seizures, hemiparesis | MRI, CT, histopathology, culture | Antifungal therapy, surgical management | 44.44% mortality |
| Hiraga *et al.* | 2017 | Japan | 1 | 21 | Female | None | Headache, fever, appetite loss, aseptic meningitis symptoms | MRI, CSF analysis, serum and CSF tests | Conservative therapy initially, then voriconazole and corticosteroids as the condition worsened | Good clinical recovery, no residual signs after 6 months |
| Panda *et al.* | 2017 | India | 1 | 25 | Female | None | GTCS, headache, proptosis, ophthalmoplegia, increased intracranial pressure | CT, MRI, MRS, histopathology, serology | Craniotomy, antiepileptic therapy, antifungal therapy | Clinical condition improved then deteriorated; continued on reduced antifungal therapy |
| Wang *et al.* | 2017 | China | 1 | 11 months | 1 Female | None | Convulsion, coma, fever, emesis | CT, MRI, bronchoscopy | Voriconazole, ceftriaxone | Improved, discharged after 4 weeks of treatment |
| Beraldo *et al.* | 2015 | Brazil | 1 | 59 | Male | None | Seizure, headache | MRI | Surgery, followed by complications | Stable, no antifungal needed |
| Mohammadi *et al.* | 2015 | Iran | 1 | 11 | 1 Male | None | Weakness after seizure, lung nodules | MRI, surgery, lung CT | Voriconazole, surgery | Initially improved, recurrence treated with second surgery |
| Ouyang *et al.* | 2015 | China | 1 | 55 | Female | None | Headache, visual acuity loss | MRI, hormonal tests | Surgery, voriconazole, Caspofungin | Symptoms improved, no recurrence |
| Tripathy *et al.* | 2015 | India | 1 | 5 months | Female | None | Progressive increase in head size, vomiting, delayed milestones | CT | Endoscopic third ventriculostomy (unsuccessful), ventriculoperitoneal shunt, voriconazole | Improved, active at follow-up |
| Bokhari *et al.* | 2014 | Saudi Arabia | 5 | 13-36 | 5 Female | Immunocompetent | Headache, seizures, neurological deficits | MRI, CT, histopathology, culture | Liposomal Amphotericin B, voriconazole, Itraconazole | Varies; some stable, one death within 2 weeks |
| Lee *et al.* | 2012 | Korea | 1 | 73 | Male | None | Headache, visual deterioration, ptosis | MRI, intra-operative biopsy | Antifungal drugs (Amphotericin-B, voriconazole) | Died from cerebral ischemia and swelling |
| Thakar *et al.* | 2012 | India | 1 | 10 months | Male | None | Hemiparesis, irritability | MRI | Surgery, Stereotactic resection | Improved, slight hemiparesis |
| Kose *et al.* | 2011 | Turkey | 1 | 23 | Female | None | Headache, blurred and double vision | MRI, biopsy | Endoscopic nasal operation, voriconazole | Lesion disappeared, voriconazole discontinued |
| Mollahoseini *et al.* | 2010 | Iran | 1 | 29 | Male | None | Focal seizures, MRI showing multiple lesions | MRI, biopsy, histological evaluation | Amphotericin B, itraconazole | Initial recovery followed by neurological decline, improvement with corticosteroids |
| Curone *et al.* | 2009 | Italy | 1 | 44 | 1 Female | None | Fever, headache, nuchal rigidity | MRI, CT, lumbar puncture | Amphotericin B, surgery | Died |
| Wasay *et al.* | 2009 | Pakistan | 25 | N.A. | 17 Male, 8 Female | Immunocompetent | Brain abscess | Clinical, MRI, Histopathology | Itraconazole, Amphotericin B | Overall mortality 40%; 100% survival in preoperative antifungal therapy group compared to 29% in postoperative group. |
| Dhamija *et al.* | 2008 | UK | 1 | 59 | Male | None | Mild proptosis, headache, sensory anomalies, vision changes | MRI, histopathology, serology | Craniotomy, antifungal therapy | Good recovery |
| Siddiqui *et al.* | 2008 | UK | 1 | 27 | Female | None | Headache, hemiparesis, seizures, coma | MRI, CT, biopsy | Amphotericin B, Mannitol | Died due to disease progression |
| Ducolin *et al.* | 2008 | Italy | 1 | 53 | Male | Pneumonia, immunocompetent | Respiratory and neurological deterioration | CT, MRI | N.A. | Died following rapid deterioration |
| Fuchs *et al.* | 2006 | Germany | 1 | Newborn | Male | None | Respiratory distress, pulmonary haemorrhage, pulmonary hypertension | Echocardiography, MRI | Mechanical ventilation, nitric oxide, surfactant, hydrocortisone, antibiotics | Died on day 28 |
| Iplikcioglu *et al.* | 2004 | Turkey | 1 | 42 | Male | None | Headache, visual disturbances, suspected sinusitis | MRI, histopathology, cultures | Transsphenoidal surgery, antifungal therapy | Recovered with normal pituitary function and no symptoms at 2-year follow-up |
| Kowacs *et al.* | 2004 | Brazil | 1 | 26 | 1 Male | None | Headache, fever, meningismus | MRI, CSF analysis | Broad-spectrum antibiotics, antifungals | Improved then deteriorated, died after 56 days |
| Siddiqui *et al.* | 2004 | Pakistan | 25 | 14-74 | 23 Male, 2 Female | None | Sinonasal aspergillosis, intracranial extension | Histopathology, CT, MRI | Surgical debridement, Amphotericin B, Itraconazole | 28% mortality, various outcomes from good recovery to moderate disability |
| Nadkarni *et al.* | 2003 | India | 1 | 32 | 1 Male | None | Epistaxis, headache, convulsions | CT, MRI, surgery | Surgery, no detailed antifungal treatment mentioned | Died from complications of surgery and aspergillosis |
| Watson *et al.* | 2002 | UAE | 1 | 25 | Female | None | Progressive bi-frontal headache, epistaxis, disorientation, short-term memory issues, third nerve palsy | MRI, ENT examination, biopsies | Liposomal amphotericin B, rifampicin, dexamethasone, GMCSF, 5-flucytosine, γ-interferon | Marked clinical improvement, maintained on itraconazole for 12 months |
| Murthy *et al.* | 2000 | India | 21 | N.A. | N.A. | None | Cranial neuropathy, cavernous sinus syndrome, orbital apex syndrome, intracranial space-occupying lesions | Biopsy, culture, CT | Surgical excision, amphotericin B (1 mg/kg/day) for 6–12 weeks, 5-fluorocytosine in some cases | One died postoperatively, five recurrences, other outcomes N.A. |

**Table 2.** Summary of all studies reporting intracranial aspergillosis in immunocompromised patients or those with immune-affecting underlying comorbidities since 2011 AD.

| Author | Year of Publication | Country | Patient Number | Age | Gender | Underlying Conditions | Clinical Presentation | Diagnostic Method | Treatment | Outcome |
| --- | --- | --- | --- | --- | --- | --- | --- | --- | --- | --- |
| Yang *et al.* | 2024 | China | 1 | 64 | Male | Diabetes, chronic mastoiditis | Chronic ear pain, purulent discharge, headaches, recurrent mastoiditis | CT, MRI, CSF mNGS, lab tests | Tympanotomy, tympanoplasty, IV antibiotics, antifungal therapy | Improved after adjusted antifungal therapy; discharged on day 37 |
| Bętkowska-Prokop *et al.* | 2024 | Poland | 1 | 62 | Male | Kidney transplantation | Respiratory failure, neurological symptoms | CT, MRI, biopsy | Voriconazole, Isovuconazole, Amphotericin B | Stable with long-term antifungal therapy |
| Amirizad *et al.* | 2023 | Iran | 1 | 11 | Female | COVID-19 | Abdominal pain, vomiting | MRI, brain biopsy | Liposomal Amphotericin B, Caspofungin | Died on day 54 post-admission |
| Samantaray *et al.* | 2023 | India | 4 | 25-55 | 3 Male, 1 Female | Diabetes, liver disease, none | Altered sensorium, seizures, fever, hemiparesis | MRI, CSF galactomannan | Voriconazole, supportive treatment | 3 died, 1 improved |
| Pei *et al.* | 2023 | China | 1 | 46 | Male | Hemifacial spasm, cerebral infarction, hypertension | Dizziness, significant growth of occipital lobe occupation | MRI, histopathology, high throughput sequencing | Surgical resection, no antifungal therapy post-discharge | Discharged without complications |
| Won *et al.* | 2023 | USA | 1 | 81 | Male | Hypertension, diabetes, chronic kidney disease, sinusitis | Fall, headache, ocular pain, diplopia, proptosis, sinus disease, epidural abscess | CT, MRI, surgical findings | IV antibiotics, endoscopic sinus surgery, antifungal therapy, multiple surgical interventions | Discharged |
| Yang *et al.* | 2022 | China | 1 | 47 | Male | AIDS | Vision issues, facial anhidrosis | MRI, CSF analysis, next-generation sequencing | Voriconazole, change in ART | Improved, ghosting vision and anhidrosis relieved |
| Janardan *et al.* | 2022 | USA | 1 | 74 | Female | Diabetes, hypertension, hyperlipidemia, arthritis | Generalized weakness, lack of appetite, severe headaches, eye pain | MRI, CT, biopsy, culture | Voriconazole, Amphotericin B, Keppra, Tylenol | Died due to complications |
| Paul *et al.* | 2022 | India | 12 | 48 | 6 Male, 6 Female | Post-COVID-19, Diabetes | Headache, nasal congestion, vision loss, facial pain, swelling | Imaging, KOH mount, culture, histopathology | Liposomal Amphotericin B, Posaconazole, endoscopic debridement | 2 died, 10 improved |
| Creuzet *et al.* | 2021 | France | 1 | 68 | Male | Chronic lymphocytic leukemia | Left hemiparesis, motor and sensory disturbance | CT, biopsy, culture | Voriconazole | Complete neurological recovery |
| Grüter *et al.* | 2021 | Switzerland | 1 | 58 | Female | Kidney transplantation, thyroid cancer | Generalized tonic-clonic seizures | CT, MRI, biopsy | Voriconazole, Amphotericin B, Isavuconazole | Discharged |
| Lange *et al.* | 2021 | Germany | 10 | 45-83 | 6 Male, 4 Female | Various, including immunosuppression | Paresis, visual impairment, headache, cranial nerve paresis | MRI, biopsy | Voriconazole, Amphotericin B, Caspofungin, surgical interventions | 60% mortality, 40% survived |
| Zeng *et al.* | 2020 | China | 1 | 37 | Male | ALL, stem cell transplant | Hemiparalysis, fever, seizures | MRI, CSF analysis, next-generation sequencing | Voriconazole, bladder surgery | Stabilized, no recurrence of aspergillosis |
| Amanati *et al.* | 2020 | Iran | 1 | 1.5 | Male | Anemia and thrombocytopenia | Bruising, fever, neutropenia, respiratory symptoms | CT, bronchoalveolar lavage, culture | Voriconazole, Caspofungin | Died from complications after prolonged hospital stay |
| Zhang *et al.* | 2020 | China | 1 | 54 | Male | Chronic hepatitis B | Chronic headache worsened by cold | MRI, CSF analysis, nanopore sequencing | Voriconazole | Improved after treatment, normal CSF on follow-up |
| Le *et al.* | 2020 | USA | 1 | 74 | Male | Chronic lymphocytic leukemia, COPD, atrial fibrillation, sarcoidosis | Imbalance, headache, confusion | MRI, craniotomy biopsy | Voriconazole | Complete resolution of CNS lesion |
| Memória *et al.* | 2020 | Brazil | 1 | 45 | Male | Chronic sinusitis, cocaine use, smoking | Headache, transient aphasia, seizures | CT, MRI, biopsy | Amphotericin B, Itraconazole | Clinical improvement |
| Mantero *et al.* | 2020 | Italy | 1 | 55 | Female | Dermatomyositis | Hemiparesis, hypoesthesia, respiratory failure | MRI, CSF analysis | Voriconazole, Amphotericin B | Died from respiratory failure and fungal co-infection |
| Kulanthaivelu *et al.* | 2020 | India | 1 | 39 | Male | Diabetes | Weakness, headaches, seizure | MRI, biopsy | Voriconazole | Stable with ongoing treatment, no headache recurrence |
| Leroy *et al.* | 2020 | France | 1 | 61 | Male | Hypertension, gout, controlled diabetes | Decreased visual acuity, diplopia, no exophthalmia | MRI, biopsy, PCR | Voriconazole, Amphotericin B | Stable, blind in right eye |
| Agrawal *et al.* | 2020 | India | 1 | 26 | Male | Suspected mucormycosis | Hemiparesis, brain abscess | CT, MRI, surgical biopsy | Liposomal Amphotericin B switched to voriconazole | Recovered, continued treatment for one year |
| Zhang *et al.* | 2020 | China | 4 | 50–69 | 3 Male, 1 Female | Various, including hypertension and coronary disease | Headaches, visual disturbances, seizures | CT, MRI, biopsy | Fluconazole, trans-sphenoidal debridement, voriconazole | Case 1 and 2 improved, Case 3 lost to follow-up, Case 4 died |
| El Hasbani *et al.* | 2019 | Lebanon | 1 | 56 | Male | Blindness, hypertension, diabetes | Unresponsiveness, labored breathing | MRI, CT, biopsy, culture | Voriconazole | Improved, no complaints at follow-up |
| Rouzaud *et al.* | 2019 | France | 2 | 39, 21 | 1 Female, 1 Male | Chronic lymphocytic leukemia, chronic granulomatous disease | Headache, fever, seizures | MRI, biopsy, culture | Amphotericin B, Voriconazole, Caspofungin, Isavuconazole | Improved, ongoing therapy |
| Liu *et al.* | 2019 | USA | 1 | 53 | Female | Glioblastoma multiforme | Productive cough, new brain lesion | MRI, biopsy | Voriconazole, needle drainage | Stabilized condition |
| Safavi *et al.* | 2019 | Iran | 1 | 12 | Male | Liver transplantation | Loss of consciousness, hemiparesis | MRI, biopsy | Voriconazole, Amphotericin B | Died after 50 days |
| Garcia-Giraldo *et al.* | 2019 | Colombia | 2 | 42, 76 | 2 Female | Obesity, bariatric surgery, hypothyroidism, chronic dacryocystitis | Bilateral exophthalmos, headache, eye pain | MRI, surgery | Amphotericin B, voriconazole | Discharged with full recovery |
| Alsulaiman *et al.* | 2019 | Saudi Arabia | 1 | 28 | Female | Pregnancy | Eyelid swelling, orbital mass, neurological symptoms | MRI, biopsy | Voriconazole, surgery | Bedridden, long-term antifungal treatment |
| Vanfleteren *et al.* | 2018 | Belgium | 1 | 65 | Female | Breast cancer history | Productive cough, dyspnea, weight loss, muscle weakness | CT, MRI, biopsy | Voriconazole, multiple neurosurgical procedures for CSF drainage | Near complete recovery |
| Divyashree *et al.* | 2018 | India | 1 | 50 | Male | Diabetes, hypertension, previous pneumonia | Headache, weakness, weight loss | MRI, biopsy, culture | Voriconazole | Improved, asymptomatic at follow-up |
| Zamora *et al.* | 2018 | USA | 1 | 71 | Female | Hypertension, hypothyroidism, acoustic neuroma surgery | Persistent fever, headaches, altered mental status, seizures | MRI, CT, CSF analysis, biopsy | Voriconazole, Amphotericin B, Colistin, Vancomycin | Improved, transferred for rehabilitation |
| Şahintürk *et al.* | 2018 | Turkey | 3 | 38-61 | 2 Male, 1 Female | Liver and renal transplants | Hemiplegia, headache, altered sensorium | MRI, CT, culture | Antifungal therapy, surgical intervention | 2 stable, 1 died |
| Houssen *et al.* | 2018 | Saudi Arabia | 1 | 33 | Male | Nasal polyp, sinusitis | Headache, fever, photophobia | CT, MRI | Multiple surgeries, Amphotericin B | Died |
| Punia *et al.* | 2018 | India | 1 | 22 | Male | Occupational exposure | Headache, vision loss, ptosis | CT, MRI, surgical biopsy | Surgery,Fluconazole, Caspofungin | Ongoing antifungal treatment |
| Kural *et al.* | 2018 | Turkey | 2 | 18-21 | 2 Male | Post-transplant, leukemia | Brain stem and lobar abscesses, seizures | MRI, surgical biopsy | Local and intravenous Amphotericin B | One death, one recovery with ongoing treatment |
| Simmonds *et al.* | 2017 | UK | 1 | 68 | Female | Hypertension, stroke, rheumatoid arthritis | Dysphasia, confusion, weakness | MRI, brain biopsy | Voriconazole, Posaconazole | Improved, continued on antifungal treatment |
| Kim *et al.* | 2017 | Korea | 1 | 55 | Female | Liver transplant recipient | Nausea, vomiting, headache, visual disturbance | CT, MRI, Histopathology | Voriconazole, ganciclovir | Significant improvement, lesion reduction |
| Turki *et al.* | 2017 | Germany | 1 | 52 | Male | T-cell large granular lymphocytic leukemia, diabetes, hypertension | Fever, cephalgia, cough | CT, MRI, CSF analysis, bronchoalveolar lavage | Imipenem, Ciprofloxacin, voriconazole | Discharged, continued oral medication |
| Baeesa *et al.* | 2017 | Saudi Arabia | 12 | 17-50 | N.A. | Varied, some diabetics | Proptosis, SAH, meningitis, epilepsy, trigeminal neuralgia, chronic headache, nasal stuffiness | CT, MRI, histopathology, culture | Surgical removal of fungal disease, antifungal therapy (amphotericin B, itraconazole, voriconazole) | Most patients improved with no relapse; two deaths due to disease progression |
| Tang *et al.* | 2016 | China | 1 | 47 | Male | Alcoholic liver cirrhosis | Deterioration of consciousness, fever, respiratory failure | MRI, ELISA, PCR | Amphotericin B | Died due to rapid disease progression |
| Tan *et al.* | 2016 | UK | 1 | 5 | Male | ALL | Seizures, hemiparesis, drowsiness | MRI, biopsy | Voriconazole, liposomal Amphotericin, surgical drainage | Resolution of hemiparesis |
| Muraoka *et al.* | 2016 | Japan | 1 | 56 | 1 Male | Paranasal sinusitis | Fever, headache, abducens nerve paralysis | PET, CT, MRI, surgery | Steroids, voriconazole, surgical resection | Deteriorated, underwent extensive surgery, GOS score 3 at 6 months |
| Neil *et al.* | 2016 | USA | 1 | 69 | 1 Male | Potential environmental exposure | Vision loss, headache | MRI, endoscopic surgery | Amphotericin lipid complex, voriconazole | Continued progression, further surgeries required |
| Al-Maskari *et al.* | 2016 | Oman | 1 | 12 | Male | Previously healthy | Sudden-onset headache, loss of consciousness, no fever or seizures | CT, MRI, histopathology | Craniotomy, resection, intravenous and oral voriconazole | Improved; resolution of lesion after 2 years of treatment |
| Sathyapalan *et al.* | 2016 | India | 1 | 35 | Female | Sarcoidosis, chronic steroids | Fever, paraparesis | MRI, spinal surgery, biopsy | Voriconazole, lipid complex amphotericin B | Improved, and transitioned to long-term rehabilitation |
| Wang *et al.* | 2016 | China | 8 | 22-64 | 5 Male, 3 Female | Various | Headache, impaired vision, ocular dyskinesia, fever | MRI, biopsy, CSF culture | Voriconazole, Fluconazole, Itraconazole, Amphotericin B | 5 died, 3 survived |
| Luo *et al.* | 2015 | China | 1 | 43 | Male | AIDS | Headache, blurred vision | MRI | Surgery, voriconazole | Neurologic deficits resolved |
| Sadarangani *et al.* | 2015 | UK | 1 | 3 | Male | ALL | Fever, leg pain, CNS lesions | MRI, biopsy | Antifungal therapy, chemotherapy adjustment | Excellent recovery, continued remission of leukemia |
| Liu *et al.* | 2015 | China | 1 | 64 | Male | Diabetes | Limb weakness, fever, headaches | MRI, Histopathology | Voriconazole | Died on hospital day 27 |
| Li *et al.* | 2015 | USA | 1 | 50 | Female | Diabetes, otomastoiditis | Headache, confusion, speech difficulty | MRI, angiography | Amphotericin B locally, antifungal therapy continued post-surgery | Lesion resolved, died 2.5 years later from unrelated causes |
| Sun *et al.* | 2015 | China | 1 | 60 | 1 Male | History of tuberculosis | Headache, weakness in right lower limb, seizure | MRI, surgical resection | Surgical treatment, voriconazole, broad-spectrum antibiotics | Died from unrelated cause 7 days post-surgery |
| Segundo *et al.* | 2014 | Brazil | 1 | 55 | Male | Diabetes, leprosy | Confusion, fever, hemiparesis | MRI, biopsy | Liposomal Amphotericin B, surgery | Improved, required extensive management |
| Bao *et al.* | 2014 | China | 1 | 42 | Male | Post-surgery for meningioma | Surgical site infection, pus, increased intracranial pressure, seizures | CT, MRI, surgical findings, lab tests | Multiple surgeries, cephalosporin, drainage, anti-epileptic, fluconazole, voriconazole | Died 1.5 years later from recurrent infection |
| Spapen *et al.* | 2014 | Belgium | 10 | 44-73 | 5 Male, 5 Female | Various, ICU patients | Refractory fever, neurological symptoms, respiratory failure | CT, brain biopsy, autopsy | Voriconazole, Amphotericin B, Caspofungin | 9 died, 1 survived |
| Bhatt *et al.* | 2013 | India | 1 | 72 | 1 Male | Chronic suppurative otitis media | Otorrhoea, headache, facial palsy | MRI, surgery, endoscopic drainage | Amphotericin B, voriconazole, antibiotics | Improved, managed with ongoing antifungal and antibiotic treatment |
| Lee *et al.* | 2013 | Korea | 1 | 48 | Male | Diabetes | Blurred vision, headache | MRI, biopsy | Amphotericin B, voriconazole | Visual symptoms improved, no recurrence |
| Vijayvargiya *et al.* | 2013 | USA | 1 | 68 | Female | Kidney transplant, diabetes mellitus, hypertension | Temporal hemianopsia, ocular ptosis | MRI, biopsy | Voriconazole, Hydrocortisone | Died due to ischemic stroke |
| Sidani *et al.* | 2013 | USA | 1 | 14 | Female | Post-intestinal transplant, PTLD | Lethargy, fever, headache | CT, MRI, open biopsy | Antifungal therapy (voriconazole, Amphotericin B) | Ongoing, improved post-antifungal therapy |
| Verma *et al.* | 2013 | India | 1 | 45 | Male | Previous pulmonary tuberculosis | Vision loss, headache, vomiting | MRI, surgery | Voriconazole | Died after acute deterioration of consciousness |
| Kim *et al.* | 2013 | Korea | 1 | 24 | Male | Type 1 diabetes, H1N1 influenza | Decreased mental status, respiratory failure, brain abscesses | MRI, CT, biopsy, culture | Voriconazole, Amphotericin B, Acyclovir, Amantadine | Improved, right hemiparesis persisted |
| Kourkoumpetis *et al.* | 2012 | USA | 14 | 31-71 | 8 Female, 6 Male | Hematologic malignancies, diabetes, COPD, transplantation | Fever, focal neurologic abnormalities, seizures, headaches | MRI, CT, biopsy | Voriconazole, Amphotericin B, Micafungin | 8 died, 6 improved |
| Cherian *et al.* | 2012 | UK | 1 | 33 | Male | Orthotopic liver transplant, acetaminophen overdose | *Aspergillus* pneumonia, hemoptysis, orbital edema | CT, BAL culture | Voriconazole, Amphotericin B | Improved, moderate renal impairment |
| Choi *et al.* | 2012 | South Korea | 1 | 72 | Female | Steroid injection, diabetes | General weakness, poor oral intake, multiple cavitary nodules in lungs | CT, MRI, biopsy | Amphotericin B, voriconazole | Died from respiratory failure |
| Kim *et al.* | 2012 | Korea | 1 | 46 | 1 Female | Systemic lupus erythematosus (SLE) | Generalized edema, headache, cough | MRI, CT, biopsy | Amphotericin B, cyclophosphamide, steroids | Initially improved, complicated by epistaxis and sinus issues |
| M. Björkholm | 2011 | Sweden | 1 | 59 | Male | Pre-B ALL | Neutropenia, fever | CT, MRI | Surgery, voriconazole | Recovery, continued chemotherapy |

**Table 3.** Summary of all studies reporting intracranial aspergillosis in immunocompromised patients or those with immune-affecting underlying comorbidities from 2000 – 2010 AD.

| Author | Year of Publication | Country | Patient Number | Age | Gender | Underlying Conditions | Clinical Presentation | Diagnostic Method | Treatment | Outcome |
| --- | --- | --- | --- | --- | --- | --- | --- | --- | --- | --- |
| Patiroglu *et al.* | 2010 | Turkey | 1 | 16 | Male | Chronic granulomatous disease suspected | Seizures, edema | MRI, culture | Surgical removal, Antifungal therapy, Interferon-γ | Improved, lesion regression after 3 months |
| Koshy *et al.* | 2010 | USA | 1 | 71 | Female | Diabetes | New onset of seizures, headache | MRI | Partial resection, voriconazole | No evidence of disease post-treatment |
| Hidron *et al.* | 2009 | USA | 1 | 28 | Male | AIDS, bacterial infections | Seizures, headache, fever | CT, MRI | Surgery, voriconazole | Improved, continued HIV treatment |
| Florescu *et al.* | 2009 | USA | 1 | 42 | Male | Cardiomyopathy, heart transplant | Infections, cerebral lesions | CT, MRI, Histopathology | Voriconazole, Caspofungin, surgery | Died 114 days post-primary transplant |
| Almutairi *et al.* | 2009 | Canada | 1 | 51 | Male | Chronic lymphocytic leukemia, bone marrow transplantation | Arm weakness and altered mental status | MRI, biopsy, culture | Fluconazole, Amphotericin B | Died due to disease progression |
| Kohler *et al.* | 2009 | Germany | 1 | 34 | Female | Crohn's disease, MVTx | Headache, sinusitis | CT, MRI | Surgery, Antifungals | Improved, no recurrence |
| Hiraga *et al.* | 2009 | Japan | 1 | 74 | Male | Hypertension, cerebellar infarction | Headache, fever | MRI, CSF analysis | Intravenous Amphotericin B, Fluconazole | Headache resolved, stable disease |
| Sutton *et al.* | 2009 | USA | 1 | 18 | Male | Post-transplant | Vocal cord paralysis, swallowing difficulty | MRI, histopathology | Intracavitary and intravenous Amphotericin B | Died due to multiorgan failure 7 days post-operation |
| Turgut *et al.* | 2008 | Turkey | 1 | 22 | Female | Aplastic anemia, prolonged steroid therapy | Headache, generalized weakness, mental confusion, hemiparesis | MRI, CT, biopsy, culture | Amphotericin B, Itraconazole, antiepileptic, antiedema therapy | Improved, continued on itraconazole |
| Azarpira *et al.* | 2008 | Iran | 1 | 49 | Female | Diabetes, chronic sinusitis | Headaches, anosmia, confusion | CT, craniotomy, histopathology | Amphotericin B | Died postoperatively |
| Srinivasan *et al.* | 2008 | India | 3 | 40-50 | 2 Male, 1 Female | Diabetes, chronic sinusitis | Visual disturbances, acute stroke, proptosis | CT, MRI, serology for HIV | Bifrontal craniotomy, medial orbitotomy, transnasal transsphenoidal approach, oral itraconazole | Improved motor power, vision not recovered, no recurrence |
| Norlinah *et al.* | 2007 | Malaysia | 1 | 45 | Male | Diabetes, hypertension | Right-sided weakness, aphasia | MRI, histopathology | Amphotericin B (discontinued), oral antifungal therapy | Died 10 days after diagnosis |
| Yoon *et al.* | 2007 | Korea | 1 | 59-72 | 1 Male,2 Female | Diabetes, hypertension, sinusitis | Headaches, vision loss, retro-orbital pain, proptosis | CT, MRI, Biopsy | Surgery, Amphotericin B, voriconazole, Itraconazole | Two had improvement but with no light perception, one died |
| Kroot *et al.* | 2007 | Netherlands | 1 | 58 | Male | Rheumatoid arthritis | Headache, vision loss, papilloedema | MRI, histological examination | Surgery, antifungal therapy | No further headaches noted |
| Sudaram *et al.* | 2007 | India | 2 | 22, 59 | 2 Male | Spinal anesthesia, diabetic, hypertensive, hypothyroid | Fever, headache, vomiting, hydrocephalus; nasal stuffiness, drowsiness, headache, fever | MRI, CSF analysis, autopsy | Antibiotics, supportive care, antituberculous drugs, steroids | Case 1 died from brain stem hemorrhage; Case 2 died from unrelated causes |
| Marinovic *et al.* | 2007 | Croatia | 1 | 65 | Male | Trauma, suspected meningitis | Meningeal signs, seizures, hemiparesis | CT, surgery, Histopathology | Antifungal therapy | Improved, managed meningitis and abscesses |
| Elter *et al.* | 2006 | Germany | 1 | 29 | Female | Chronic polyarthritis, prednisone | Headaches, eyelid swelling, forehead numbness | MRI, biopsy, culture | Voriconazole, Amphotericin B, Caspofungin, Fosfomycin, Floxacillin | Stable, no clinical or neurological impairment |
| Tsai *et al.* | 2006 | China | 1 | 48 | Female | Psoriasis, cervical carcinoma | Invasive pulmonary aspergillosis, fever, cough, shortness of breath | Bronchoscopic culture and biopsy, CT, MRI | Amphotericin B, itraconazole, respiratory support | Died of unrelated cause |
| Middelhof *et al.* | 2005 | USA | 4 | 6-16 | N.A. | Acute myelocytic leukemia | CNS aspergillosis | CT, MR, stereotactic surgery | Stereotactic resection of aspergillomas, amphotericin B, voriconazole | Improved survival with neurological recovery, one death due to leukemia |
| Sterba *et al.* | 2005 | Czech Republic | 1 | 16 | Female | ALL | Febrile neutropenia, space-occupying brain lesion | MRI, histopathology | Amphotericin B, stereotactic biopsy, intracavitary cAmpho B, voriconazole | Significant healing of abscess, leukemia in remission |
| Vidal *et al.* | 2005 | Brazil | 1 | 26 | Male | HIV, previous infections | Fever, cough, dyspnea, seizures | CT, surgical biopsy | Amphotericin B, Itraconazole | Died from pneumonia three months after admission |
| Giacchino *et al.* | 2005 | Italy | 4 | 2-14 | 4 Male | Lenz syndrome, meningioma, Burkitt's lymphoma | Fever, functional impairment, sinusitis, seizures | CT, MRI, nasal samples | Surgery, Amphotericin B, Itraconazole, Flucytosine, Caspofungin | Significant recovery |
| Lacerda *et al.* | 2005 | Portugal | 1 | 52 | Female | Multiple myeloma, SCT | Fever, GI symptoms | MRI | Surgery, Amphotericin B | Died 7 days post-surgery |
| Bethell *et al.* | 2004 | UK | 1 | 2.5 | Female | ALL | Infection symptoms | MRI, Ultrasound | Multiple antifungals, Surgery | Died of unrelated casue |
| Merseburger *et al.* | 2004 | Germany | 1 | 53 | Male | Bladder cancer | Macrohematuria, suspected metastases | CT, Histopathology | No antifungal treatment post-surgery due to patient refusal | Died from unrelated cause |
| Ohmagari *et al.* | 2004 | USA | 13 | 20-76 | 11 Male, 2 Female | Solid tumors, not neutropenic | Various clinical signs of invasive aspergillosis | Mycologic assessments, CT, MRI | Surgical resection, antifungal therapy (Amphotericin B, Itraconazole) | 23% mortality at 6 weeks |
| Ho *et al.* | 2004 | Belgium | 1 | 48 | Male | Tuberculosis, liver cirrhosis | Pneumonia, respiratory failure, neurological symptoms | Broncho-alveolar lavage, CT, MRI | Amphotericin-B, 5 Fluorocytosine, supportive care | Died following complications |
| Tattevin *et al.* | 2004 | France | 8 | 46–6 | 4 Male, 4 Female | Various immunosuppressive conditions | Fever, headache, neurological symptoms, seizures | CT, MRI, lumbar puncture | Amphotericin B, flucytosine, itraconazole, surgical interventions | 5 died (brain stem herniation, neurological deterioration), 3 survived with no neurological sequelae |
| Colombo *et al.* | 2003 | Brazil | 1 | 66 | Female | Diabetes, previous cranial trauma | Frontal headache, swelling of the skull at the right temporo-parietal region, signs of osteomyelitis and a brain abscess | CT, histopathological assessment of bone tissue, culture | Craniotomy, surgical debridement, amphotericin B, caspofungin, itraconazole | Complete neurological recovery, maintained on suppressive antifungal therapy |
| Lastours *et al.* | 2003 | France | 2 | 48 | 1 Male, 1 Female | Heavy smoking, chronic alcoholism; Type 2 diabetes, Hepatitis C, HIV | Generalized seizure, fever, confusion; right-sided earache, left-sided facial palsy, fever, asthenia | CT, MRI, stereotactic biopsy, cultures | Amphotericin B, itraconazole, voriconazole | Patient 1 improved, asymptomatic at 6 months; Patient 2 died of unrelated cause |
| Antony *et al.* | 2003 | USA | 1 | 7 | Male | Traumatic brain injury | Purulent drainage from a scar, frontal lobe abscess | CT, MRI, surgical debridement, cultures | Amphotericin B, AmBisome, rifampin, extensive debridement, free flap for soft-tissue coverage, switched to oral itraconazole | Healed wounds, no residual infection at 1-year follow-up |
| Endo *et al.* | 2002 | Japan | 1 | 50 | Female | Hypertension, mild renal dysfunction | Sudden onset headache, SAH, fever post-op. | CT, MRI, angiography, CSF analysis, post-mortem microscopic examination | Clipping of AComA aneurysm, antibiotic treatment, repeat craniotomy | Died on day 40 post-op from repeated SAH and ischemic damage |
| Erdogan *et al.* | 2001 | Turkey | 1 | 45 | Female | Stage IIIA breast cancer | Developed ataxia and gait disturbance after treatment, found with right cerebellar lesion | CT, MRI | Surgical removal of cerebellar abscess, suboccipital craniectomy, AmB 1 mg/kg/d for 3 months, itraconazole 100 mg/kg/d for 1 year | Improved, no recurrence of infection after 3 months, alive and well at 2-year follow-up |
| Saulsbury *et al.* | 2001 | USA | 1 | 11 | Male | X-linked chronic granulomatous disease (CGD) | Headache, fever, diagnosed with a cerebellar abscess | MRI, surgical exploration, cultures | Itraconazole, IFN-γ, amphotericin B | Complete resolution of abscess, no recurrence in 9 years |
| Ng *et al.* | 2000 | UK | 1 | 2 | Female | ALL | Bruising, anemia, lymphadenopathy, complex partial seizure | MRI, CSF analysis, cytochemical staining | Intensification chemotherapy, cranial irradiation, AmBisome, flucytosine, GCSF | Died of unrelated causes |
| Cuccia *et al.* | 2000 | Argentina | 3 | 5-14 | 3 Male | Anaplastic ependymoma, aplastic anemia, chro1+nic renal failure | Headache, vomiting, hemiparesis, proptosis, hemiparesis, coma, seizures | CT, MRI, funduscopic exam, biopsy | Surgical resection, amphotericin B, 5-flucytosine, radiation therapy, chemotherapy | One successful recovery, one controlled hydrocephalus but persistent mutism, one death due to uncontrollable fungemia |
| Marcinkowski *et al.* | 2000 | Germany | 1 | Newborn | Male | Atrioventricular septal defect, DiGeorge syndrome | Tachycardia, dyspnea, septic shock, renal failure, periventricular hemorrhagic infarction | Clinical exam, ultrasound, autopsy | Digitalis, diuretics, various antibiotics, prostaglandin E1, catecholamines, peritoneal dialysis, hemicolectomy | Died at 24 days due to complications of underlying conditions and infections |
| Pidhorecky *et al.* | 2000 | USA | 13 | 11-77 | 10 Male, 3 Female | Hematological malignancies, one with breast cancer | Symptoms of IPA in neutropenic patients post myeloablative treatment, including cough, fever, hemoptysis | CT, BAL, clinical symptoms | Surgical resection of lung lesions, antifungal treatment (amphotericin B, itraconazole) | 5 lived, 7 died from disease progression, 1 perioperative death, and 2 postoperative deaths from complications and disseminated IPA |

Note: Tables 1-3 summarize published case reports and case series of intracranial aspergillosis in immunocompetent and immunocompromised patients from 2000 to 2024. The detailed methodology, including search strategy and inclusion criteria, is provided in the Discussion section. Abbreviations: CT: Computed Tomography; MRI: Magnetic Resonance Imaging; CSF: Cerebrospinal Fluid; GTCS: Generalized Tonic-Clonic Seizures; AIDS: Acquired Immunodeficiency Syndrome; ALL: Acute Lymphoblastic Leukemia; AML: Acute Myeloid Leukemia; COPD: Chronic Obstructive Pulmonary Disease; ART: Antiretroviral Therapy; BAL: Bronchoalveolar Lavage; SCT: Stem Cell Transplant; SAH: Subarachnoid Hemorrhage; GOS: Glasgow Outcome Scale; ICU: Intensive Care Unit; N.A.: Not Available; DM: Diabetes Mellitus; HTN: Hypertension; TB: Tuberculosis; CLL: Chronic Lymphocytic Leukemia; ESRD: End-Stage Renal Disease.
